# Supplementary material for: Synchronization in electric power networks with inherent heterogeneity up to 100% inverter-based renewable generation
Source: Nat Commun. 2022 May 5;13:2490. doi: 10.1038/s41467-022-30164-3 (PMC9072397; doi:10.1038/s41467-022-30164-3)
Supplement: Supplementary file 1 — Supplementary Information [file 41467_2022_30164_MOESM1_ESM.pdf]

# Synchronization in Electric Power Networks with Inherent Heterogeneity Up to 100% Inverter-Based Renewable Generation

Amirhossein Sajadi[1], Rick Wallace Kenyon[1,2,3], Bri-Mathias Hodge[1,2,3]
[1]Renewable and Sustainable Energy Institute at the University of Colorado Boulder, 4001 Discovery Drive, Boulder, CO 80303, USA
[2]Electrical, Computer, and Energy Engineering Department at the University of Colorado Boulder, 425 UCB, Boulder, CO 80309, USA
[3]Grid Planning and Analysis Center at the National Renewable Energy Laboratory (NREL), 15013 Denver W Pkwy, Golden, CO 80401, USA

## Supplemental Material

*Supplementary Note 1: Modelling a mechanical system of coupled mass-spring-damper*

The coupled mechanical mass-spring-damper system is a classical problem that represents a simple harmonic oscillator. Here, we use this example as a mechanical analog to describe the problem of synchronization of generators in an electric power system, as depicted in Supplementary Figure 1.

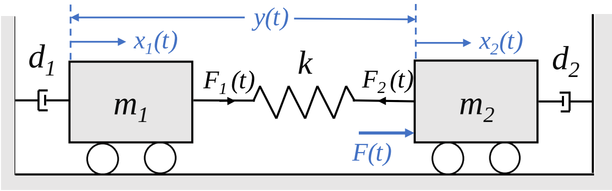


Supplementary Figure 1: Coupled mechanical mass-spring damper system with two carts, two dampers, and a spring connected between them..

The simultaneous differential equations that describe the motion of the masses in the system depicted in Supplementary Figure 1, assuming initial values equivalent to zero, are:

$$\begin{aligned} m_1\ddot{x}_1 + d_1\dot{x}_1 &= k(x_2 - x_1) \\ m_2\ddot{x}_2 + d_2\dot{x}_2 &= -k(x_2 - x_1) + F \end{aligned} \tag{1}$$

Assuming $F = 0$, in order to study the natural modes, they can be rewritten as

$$\begin{aligned} \ddot{x}_1 + \frac{d_1}{m_1}\dot{x}_1 &= \frac{1}{m_1}k(x_2 - x_1) \\ \ddot{x}_2 + \frac{d_2}{m_2}\dot{x}_2 &= \frac{k}{m_2}(x_1 - x_2) \end{aligned} \tag{2}$$

In matrix form, defining $x = \begin{bmatrix} x_1 & x_2 \end{bmatrix}^T$, they are:

$$\ddot{x} + \frac{D}{M}\cdot\dot{x} + \frac{K}{M}\cdot x = 0 \tag{3}$$

with $\frac{D}{M} = diag\{\begin{bmatrix}\frac{d_1}{m_1} & \frac{d_2}{m_2}\end{bmatrix}\}$ and $\frac{K}{M} = \begin{bmatrix}\frac{k}{m_1} & -\frac{k}{m_1}; -\frac{k}{m_2} & \frac{k}{m_2}\end{bmatrix}$. By defining $v_1 = \dot{x}_1$ and $v_2 = \dot{x}_2$, a first order state space model is created:

$$\begin{aligned} \dot{x}_1 &= v_1 \\ \dot{x}_2 &= v_2 \\ \dot{v}_1 &= -\frac{d_1}{m_1}v_1 + \frac{k}{m_1}x_2 - \frac{k}{m_1}x_1 \\ \dot{v}_2 &= -\frac{d_2}{m_2}v_2 + \frac{k}{m_2}x_1 - \frac{k}{m_2}x_2 \end{aligned} \tag{4}$$

Solving this system of differential equations yields the dynamical equations governing the speed as a function of time, $v_1(t)$ and $v_2(t)$, respectively. Then kinetic energy as a function of time can be computed by $E(t) = \frac{1}{2}\ m_1 \cdot v_1^2(t) + \frac{1}{2}\ m_2 \cdot v_2^2(t)$. Supplementary Table I summarizes the mechanical and electrical analogous variables.

Supplementary Table I: Summary of mechanical and electrical analogous variables

| **Variable** | **Mechanical System** | **Electrical System** |
|---|---|---|
| Capacitive Energy Storage | $m_i$ | $M_i$ |
| Energy Dissipator | $d_i$ | $D_i$ |
| Inductive Energy Storage | $k_{ij}$ | $Y_{ij}$ |
| Position | $x_i$ | $\delta_i$ |
| Velocity | $v_1$, $v_2$ | $\omega_1$, $\omega_2$ |
| Force | $k_{ij}\cdot(\mathrm{x}_i - x_j)$, ∀i,j | $Y_{ij}\cdot(\delta_i - \delta_j)$, ∀i,j |
| Synchronized State | $v_1 = v_2 = \cdots = \mathrm{v}_i$ | $\omega_1 = \omega_2 = \cdots = \omega_i$ |
| Kinetic Energy | $\sum_{i=1}^{n} 0.5\ m_i \cdot v_i^2$ | $\sum_{i=1}^{n} 0.5\ M_i \cdot \omega_i^2$ |

*Supplementary Note 2: Dynamic model of the generators*

Electric power systems can be modeled as a complex network with power generators, substations, and load centers constituting the nodes and the transmission lines that interconnect them the links. Generally, the behavior of power systems can be described using a set of differential-algebraic equations (DAE) equations, as in [1]:

$$\begin{aligned} \dot{x}(t) &= f(t, x(t), y(t), u(t)) \\ 0 &= g(t, x(t), y(t), u(t)) \end{aligned} \tag{5}$$

where dot notation indicates time derivative; $\dot{x} = \frac{d}{dt}x$, $t$ denotes time, $x$ is the vector of state variables, $y$ is the vector of algebraic variables, and $u$ is the vector of inputs. The vector field $f(t, x, y, u)$ describe the dynamic evolution of the state variables of the system including generation, load, and any other dynamic elements and the associated control systems. The algebraic equations determined by $g(t, x, y, u)$ describe the system constraints and the electric network that interconnects the dynamic elements [1]. In this study, we focus on the impacts of generator parameters on the system synchronization. Thus, we model the system generators [1] as dynamic elements. The transmission lines and load centers, whose contribution to inertia is relatively minimal, are modelled as algebraic

elements. The power generation technologies considered here can be primarily divided into two main categories: (1) synchronous generator-based power plants and (2) power electronics-based power plants.

*Synchronous Machine-based Power Plants:* In conventional power systems, the majority of generation facilities are equipped with synchronous generators that transform mechanical power directly into alternating current electric power. Over the past century, synchronous generators - including various types driven by disparate fuels such as coal, natural gas, nuclear fission, hydro, etc - have matured and presently offer a reliable and economical performance for the bulk generation of electricity. They have two main electromagnetic parts: (1) *Stator:* which is a set of stationary windings, sinusoidal distributed around a rotating shaft and (2) *Rotor:* which is a mechanically rotating shaft housed within the stator, that has the primary field winding, and damper windings. The damper windings on the rotor shaft help to damp out the machines oscillations induced during transient behaviors [2]. The general structure of a synchronous generator is shown in Supplementary Figure 2.

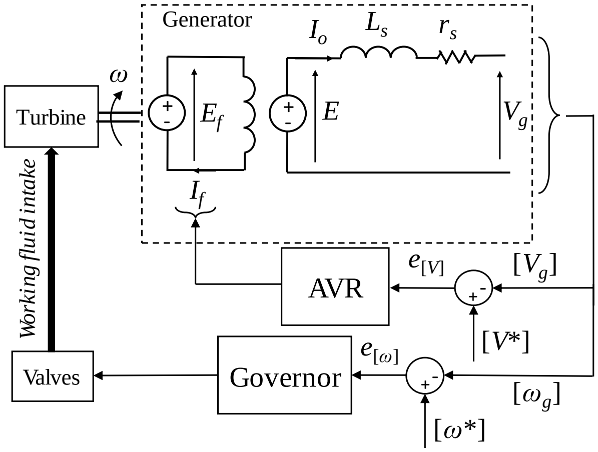


Supplementary Figure 2: Simplified synchronous generator control system.

When the loading condition on synchronous generator changes, the rotation speed, and thus the frequency changes. This change in frequency is registered by a governor, which varies mechanical power to match the electric power demand, through a feedback control process with the objective to track the frequency reference. The adjustment of mechanical power comes in three steps. First, the mechanical power adjustment is determined by the droop value, which is a linear compensator. Second, the mechanical power is adjusted accordingly by the governor, which elicits a turbine response. Third, the frequency excursion is arrested when the adjusted mechanical power applies to the shaft of the synchronous machine. This process, assuming the voltage dynamics remain ideal, is summarized in Supplementary Figure 3.

Using simplification rules for closed-loop feedback control systems, one can write a transfer function of the synchronous generator frequency response to changes of power output as:

$$\frac{\Delta\omega_{SG}}{\Delta P_e} = \frac{\frac{1}{M_m s + D_m}}{1 + \frac{1}{M_m s + D_m} \cdot \frac{1}{R} \cdot \frac{K_{TG}}{1 + T_{TG} s}} = \frac{\frac{1}{M_m}\left(s + \frac{1}{T_{TG}}\right)}{s^2 + \left(\frac{1}{T_{TG}} + \frac{D_m}{R \cdot M_m}\right)s + \left(\frac{D_m}{M_m \cdot T_{TG}} + \frac{K_{TG}}{R \cdot M_m \cdot T_{TG}}\right)} \tag{6}$$

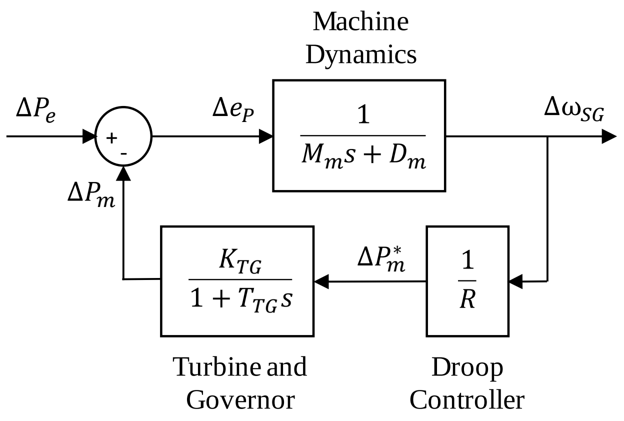


Supplementary Figure 3: Laplace block diagram of the reduced-order model for frequency response in SG

where

- $D_m$ is the mechanical damping torque of the damper windings
- $M_m$ is the mechanical inertia of all coupled, rotating parts
- $R$ is the droop gain
- $T_{TG}$ is the governor and turbine response time constant
- $K_{TG}$ is the governor and turbine response gain constant

This transfer function presents a reduced-order model for the frequency response of the synchronous generator. It should be noted that $D_m$ is a function of rotor angle ($\delta$), $D_m = f(\delta)$, though for small deviations from the synchronous speed, its variation is minimal and can be assumed a constant, $D_m(\delta) \approx D_m$ [2], [3].

The roots of the characteristic equation for the synchronous generator are calculated by

$$s_{1,2} = \frac{1}{2}\Big( - (\frac{1}{T_{TG}} + \frac{D_m}{R \cdot M_m}) \pm \sqrt{(\frac{1}{T_{TG}} + \frac{D_m}{R \cdot M_m})^2 - 4(\frac{D_m}{M_m \cdot T_{TG}} + \frac{K_{TG}}{R \cdot M_m \cdot T_{TG}})}\Big) \tag{7}$$

Looking at ($s_{1,2}$), the damping component is mainly determined by the real part of these roots, $-(\frac{1}{T_{TG}} + \frac{D_m}{R \cdot M_m})$. According to this relationship, for a synchronous generator, the components that contribute to damping are (1) the mechanical damping provided by the damper winding, (2) product of droop gain and mechanical inertial momentum, and (3) the response time constant of the governor and turbine system. Also from the transfer function, one can draw a conclusion that the pace of frequency response (the inertial component) is directly tied to the mechanical inertia ($\frac{1}{M_m}$), particularly the fast transient portion of the response because of the existence of a zero (the term $s$ in the numerator). The mathematical description of the frequency response of a synchronous generator concludes the followings: the larger the mechanical dampers, $D_m$, and/or the smaller the product of the droop gain and mechanical inertia, ($R.M_m$), and/or the smaller governor and turbine response time, $T_{TG}$, (indicating a faster response time), the larger the magnitude of the negative real part of imaginary number, which indicates a higher damping of the natural modes. The larger the mechanical inertia, ($M_m$), the slower the rate of change of frequency, a well known result in power system

dynamics.

We verified and validated this reduced-order model for synchronous generator frequency response, described in Eq. (6), using higher-order models developed in PSCAD (abbreviation of "power system computer aided design"). We used the open-source models developed by the National Renewable Energy Laboratory (NREL) and University of Colorado Boulder (CU Boulder), which are available to public for research use [4]. The mathematical description of the high-order models for synchronous generators and associated power network used in these PSCAD test cases are explained in [5]. PSCAD is an industry grade software package that operates in the electromagnetic transient (EMT) domain and is capable of capturing the fast dynamics of controllers that are generally obscured in phasor domain simulation tools. All three electrical phases are modelled with a suitable time resolution (in the microsecond range) to capture fast transients. The results of the validation are shown in Supplementary Figure 4 and corroborate that the reduced-order model is effective at approximating the frequency response of a synchronous generator.

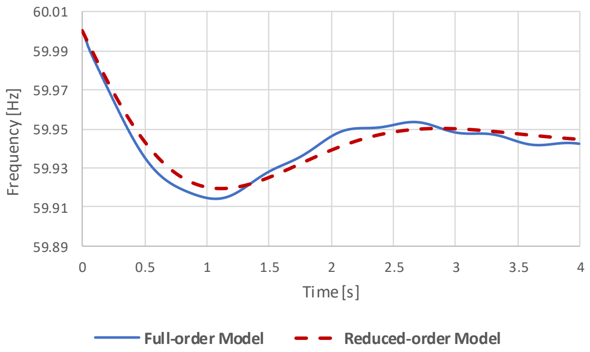


Supplementary Figure 4: Validation of the synchronous generator frequency response using the reduced-order model by comparing it with the frequency response using the high-order results obtained from the PSCAD simulation for 3.5MW load switching in the 9-bus system - the model is available open-source at [5].

The transfer function of the reduced-order frequency response model is a second-order dynamical system. Therefore, for studying synchronization, we use a generic second-order model in the form of a second-order ordinary differential equation (common harmonic oscillator model) with electric angle and speed as the dynamic variables. Using the generic model, assuming

$x_1 = \delta_{sg}$, $x_2 = \omega_{sg}$, the frequency response of a synchronous generator can be described by:

$$\begin{aligned} \dot{x}_1 &= x_2 \\ \dot{x}_2 &= M_{sg}^{-1}(P^* - P_e - D_{sg}x_2) \end{aligned} \tag{8}$$

where $p^*$ and $p_e$ are the mechanical power input and electrical power export, respectively. The $M_{sg}$ and $D_{sg}$ are inertia and damping coefficients.These coefficients represent all contributing elements to inertia and damping components. Because of the mechanical nature of energy conversion with synchronous machines, the response agility is bounded by relatively slow mechanical adjustments. The inertia and damping coefficients are constant, as they are dependent on the machine design, and can be expressed by the $D/M$ ratio for simplicity. The values of these variables are physical constraints

and are set by the machine and power plant design; during system operation, they generally cannot be changed. When subjected to a disturbance that results in mismatch between the generator mechanical power and electric power output, the electric speed and subsequently the electric angle, experience transients. We verified and validated this generic second-order model presented in Eq. 8 using the previously validated reduced-order model presented in Eq. (6). The results of the validation are shown in Supplementary Figure 5 and corroborate that the generic second-order model can effectively approximate the frequency response of the synchronous generator.

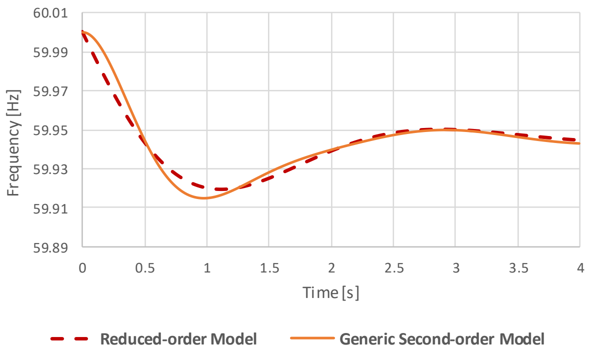


Supplementary Figure 5: Validation of the synchronous generator frequency response obtained from the generic second-order model by comparing it with the frequency response obtained from the reduced-order model, emulating a 3.5MW load switching event in the 9-bus system

.

*Power electronics-based power plants:* Power electronic inverters, which are often used to interface variable renewable energy sources with the grid, can be categorized by the primary control mode as either (i) grid-following (GFL) or (ii) grid-forming (GFM). Both classes of inverters have similar hardware components with the structure differing chiefly in the control schemes, which has a considerable impact on the interactions with the electric power grid. GFL inverters, the most commonly used class of inverters, rely on the phase locked loop (PLL) to estimate and track the frequency at the point of interconnection (POI). Typically, the control objective of these devices is to maintain a set power export regardless of varying grid conditions. If operated as a grid support device using a droop-relationship, then active power and reactive power are adjusted proportionally to frequency and voltage deviations. In contrast, GFM inverters, an emerging and promising technology as applied to the parallel operation in bulk power systems, adjust the frequency and voltage according to the power injection deviations at the POI. In other words, they are capable of supporting the grid by generating frequency independent of the grid operating conditions. An additional distinction in GFM control is the necessity of available positive headroom to perform these control objectives. Although there are variants for each of these two classes in the literature [6]–[9], this work focuses on the two most common state-of-the-art classes: (1) the GFM with either the virtual-inertia support functionality or the multi-loop droop control approach and (2) the GFL. The general structure of these technologies is shown in Supplementary Figure 6.

The GFM with virtual-inertia support functionality is also known as the virtual synchronous machine (VSM) [10], with the aim to emulate a synchronous generator frequency response to mitigate

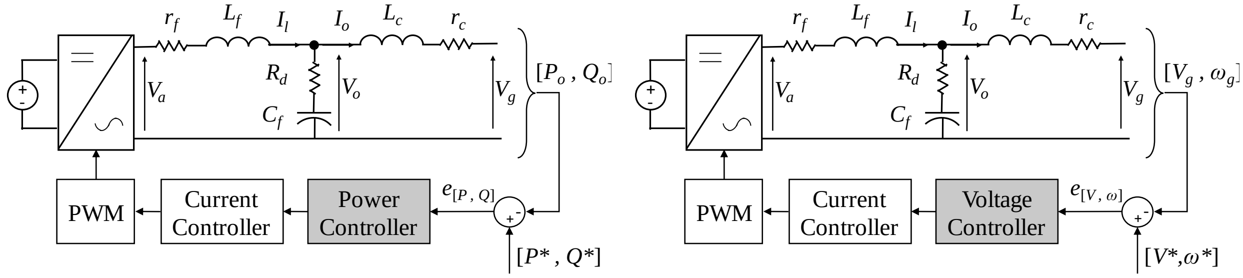


(a) GFL (b) GFM

Supplementary Figure 6: Comparative illustration of simplified GFL and GFM control systems

potential operational challenges that grid-coupled inverters may pose [11]–[18]. Subsequently, at least 11 control approaches have been proposed for the VSM [10], [11], [19]–[38], all of which are neatly summarized in [39], with the primary differences generally associated with internal decision-making and the choice of state variables. Nonetheless, all of these various approaches have a similar output as they try to emulate a synchronous generator and, thus, their frequency response can be described by the second-order differential equation used for a synchronous generator. The main distinction is that the physical variables should be replaced with their digital dual and a similar frequency response mechanism applies.

The mechanism of frequency response in the multi-droop GFM inverter adjusts the frequency set point as a function of output power deviations through an open-loop control process that leverages the frequency-droop relationship of real power flow across AC transmission systems. The adjustment of frequency comes as the following: Output power is determined by the voltage values at the two terminating points of the grid coupling inductor of the LCL filter. Denoting $E$ and $V$ the two voltage values and the value of the inductance $X_{L_c}$, the power transfer is $P_g = \frac{E \cdot V}{X_{L_c}} sin\delta$. Assuming the voltages are very close to 1 p.u. and after linearization, the change of the multi-loop GFM power output can be described by $\Delta P_g = \frac{1}{X_{L_c}} \Delta\delta cos\delta$. Since $\delta$ is a small value, $cos\delta \approx 1$, and $\Delta P_g = \frac{1}{X_{L_c}} \Delta\delta$. For frequency response, first, the power error is detected through a low-pass filter whose output is used to create the power error signal for the droop controller. Second, the droop controller determines the output frequency set point. Finally, passing the frequency set point through an integrator creates the angle of voltage at the capacitor of the LCL filter. As noted, this is an open-loop process. This process is summarized in Supplementary Figure 7.

One can write a transfer function of the multi-loop GFM frequency response as a function to changes of power demand as:

$$\frac{\Delta\omega_{GFM}}{\Delta P_e} = \frac{R}{T_{\omega_c} s + 1} = \frac{\frac{R}{T_{\omega_c}}}{s + \frac{1}{T_{\omega_c}}} \tag{9}$$

where

- $R$ is the droop gain, and
- $T_{\omega_c}$ is the low-pass filter time constant, the reciprocal value of cut-off frequency ($\omega_c$),

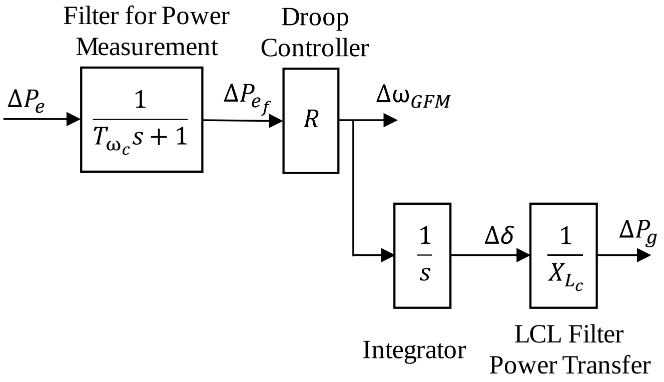


Supplementary Figure 7: Laplace block diagram of the reduced-order model for frequency response in multi-loop GFM

The characteristic equation for the multi-loop GFM inverter is a first-order polynomial whose singular root is

$$s_1 = -\frac{1}{T_{\omega_c}} = -\omega_c \tag{10}$$

which is the reciprocal value of the time constant of the low-pass power measurement filter, equal to the cut-off frequency. Knowing the cut-off frequency is relatively high $\omega_c = \frac{1}{T_{\omega_c}}$ ($\omega_c = \frac{1}{T_{\omega_c}} > 100$ rad/sec) [40], the only root of this equation is well to the left hand side of the imaginary axis and always real, and thus, the multi-loop GFM inverter is safe be assumed asymptotically stable.

The transfer function indicates that the frequency response of multi-loop GFM is first-order. The pace of response (the inertial component) is directly determined by the cut-off frequency of the low-pass power measurement filter, $\omega_c$, and the droop gain $R$. The settling value is determined by the droop gain, $R$; the higher the cut-off frequency or larger the droop gain, the faster the frequency deviates, and the smaller the droop gain, the smaller the frequency deviation.

We verified and validated this reduced-order model for GFM frequency response, described in Eq. (9), using the high-order models developed in PSCAD. We used the very same open-source models developed by the NREL and University of Colorado Boulder (CU Boulder) [4] that previously were used to verify the synchronous generator reduced-order model. The mathematical description of the high-order models for the GFM inverter and the associated power network used in this PSCAD test case are detailed in [5]. The results of validation are shown in Supplementary Figure 8 and indicate that the reduced-order model can suitably approximate the frequency response of the GFM.

The transfer function of the reduced-order frequency response model for the multi-loop droop GFM yields a first-order system which can be approximated by order-reduction of a second-order system, while the transfer function for the VSM-GFM yields a second order model. Therefore, for studying synchronization, we use a generic second-order model in the form of a second-order ordinary differential equation (common harmonic oscillator model) with electric angle and speed as the dynamic variables. Using the generic model, assuming $x_1 = \delta_{pe}$, $x_2 = \omega_{pe}$, the frequency response of GFM can

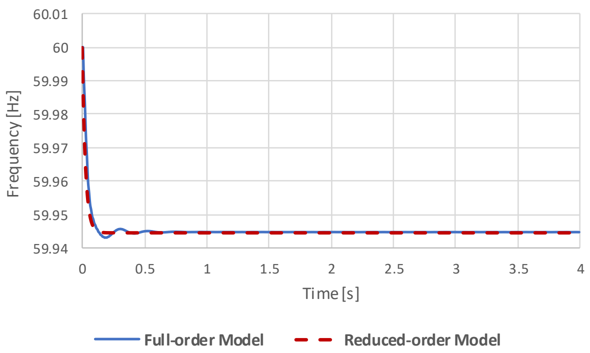


Supplementary Figure 8: Validation of the GFM frequency response obtained from using the reduced-order model obtained from the analytical model by comparing it with the frequency response using the full-order results obtained from the PSCAD EMT simulation for a 3.5MW load switching event in the 9-bus system

be described by

$$\begin{aligned}\dot{x}_1 &= x_2 \\ \dot{x}_2 &= M_{pe}^{-1}(P^* - P_e - D_{pe}x_2)\end{aligned} \tag{11}$$

where $p^*$ and $p_e$ are the power output setpoint and actual power export, respectively. The $M_{pe}$ and $D_{pe}$ are the equivalent inertia and damping coefficients; when $\{M >> 0\}$ the VSM control is employed and for $\{M \approx 0 \wedge M \neq 0\}$ the control mode is the multi-loop droop class of GFM (which would result in order reduction to the first-order). These coefficients represent all contributing elements to inertial and damping component of frequency response In power electronics-backed generation units, because of the non-mechanical nature of energy conversion with DC sources, the response agility is primarily bounded by the digital controller process time, which could be very quick, and can be modified in an adaptive manner and in real-time, except for the physical value of the grid-coupling inductance. Therefore, unlike synchronous generators, the inertia and damping coefficients in GFM are independent of each other. The power setpoint defines the amount of power that is commanded to be released through the semi-conductor switches. This model implies that following a disturbance that results in mismatch between the GFM power setpoint and power output, electric angle can begin to oscillate. In a stable operation, these oscillations should be damped out within a relatively short time following their inception - within seconds.

We verified and validated this generic second-order model presented in Eq. 11 for multi-loop GFM using the previously validated reduced-order model presented in Eq. (6). The synchronous generator model validation accounts for the VSM-GFM model validation. The results of the validation are shown in Supplementary Figure 9 and show that the generic second-order model can effectively approximate the frequency response of a multi-loop GFM.

The GFL, on the other hand, does not contribute to the system electric angle construction. The GFL does not produce any constituting voltage frequency and instead follows the frequency at the POI. It estimates the frequency at the POI using a PLL and through a linear relationship (droop) it adjusts its power export by acting as a current source. Therefore, the its governing equation can

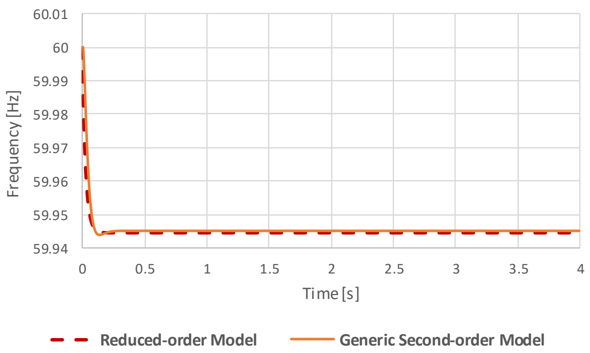


Supplementary Figure 9: Validation of the multi-loop GFM frequency response obtained from the generic second-order model by comparing it with the frequency response obtained from the reduced-order model, emulating 3.5MW load switching in the 9-bus system

be given by:

$$0 = P^* - P_e \tag{12}$$

where $p^*$ and $p_e$ are the power setpoint and power export. Effectively, the GFL can be modeled as a negative load.

*Supplementary Note 3: Linear model of electric power network*

To obtain the Jacobian of the electric power network of interest in this study, first, let us consider the operation at a stable equilibrium where the generator power setpoints and electric power exports are equal, $P^* = P_e$, for both the GFM and Synchronous generators. Following a disturbance, there will be a mismatch between these known as the acceleration power, defined by $P = P^* - P_e$ as pertaining to changes in generators' power output. Inspired by Machowski's [2] analysis of power networks with only synchronous generators considered, linearization of the governing equations for the GFM and Synchronous machines (the linearization introduces the linear difference operator, $\Delta$) interconnected at the $i$-th node (8) and (11) yields:

$$\begin{aligned} \Delta\dot{\delta}_i &= \Delta\omega_i \\ \Delta\dot{\omega}_i &= \frac{1}{M}\Delta P_i - \frac{D}{M}\Delta\omega_i \end{aligned} \tag{13}$$

where $\Delta P_i$ is the change in the generator power injection. For any node $i$, the active power injection at the node with voltage of $V_i = |V_i|\angle\delta_i$ can be expressed by power-flow equation as [2]:

$$P_i = V_i^2 \cdot G_{ii} + \sum_{j=1;j\neq i}^{n} V_i \cdot V_j \cdot [B_{ij} \cdot sin(\delta_i - \delta_j) + G_{ij} \cdot cos(\delta_i - \delta_j)] \tag{14}$$

where $Y_{ij} = G_{ij} + j\ B_{ij}$ here are the effective admittance values obtained from Kron reduction; $G$ is the conductance and $B$ is the susceptance. The linearized form of (14) can be expressed in a matrix

form as:

$$\begin{bmatrix} \Delta P_1 \\ \vdots \\ \Delta P_n \end{bmatrix} = \begin{bmatrix} \frac{\partial P_1}{\partial \delta_1} & \cdots & \frac{\partial P_1}{\partial \delta_n} \\ \vdots & \ddots & \vdots \\ \frac{\partial P_n}{\partial \delta_1} & \cdots & \frac{\partial P_n}{\partial \delta_n} \end{bmatrix} \begin{bmatrix} \Delta \delta_1 \\ \vdots \\ \Delta \delta_n \end{bmatrix} \tag{15}$$

Equation (15) can alternatively be presented as:

$$\begin{bmatrix} \Delta P_1 \\ \Delta P_2 \\ \vdots \\ \Delta P_n \end{bmatrix} = \begin{bmatrix} H_{1,1} & H_{1,2} & \dots & H_{1,n} \\ H_{2,1} & H_{2,2} & \dots & H_{2,n} \\ \vdots & \vdots & \ddots & \vdots \\ H_{n,1} & H_{n,2} & \dots & H_{n,n} \end{bmatrix} \begin{bmatrix} \Delta \delta_{1,n} \\ \Delta \delta_{2,n} \\ \vdots \\ \Delta \delta_{n,n} \end{bmatrix} \tag{16}$$

$$\Delta P_n = H_n \; \Delta \delta_n \tag{17}$$

where $H$ is the system network Laplacian (effective admittance), an $(n \times n)$ matrix whose elements can be computed by:

$$\begin{aligned} H_{ij} &= -V_i \cdot V_j \cdot [B_{ij} \cdot cos(\delta_i - \delta_j) - G_{ij} \cdot sin(\delta_i - \delta_j)] \\ H_{ii} &= \sum_{j=1; j \neq i}^{n} V_i \cdot V_j \cdot [B_{ij} \cdot cos(\delta_i - \delta_j) - G_{ij} \cdot sin(\delta_i - \delta_j)] \end{aligned} \tag{18}$$

Replacing (17) into (13) produces:

$$\begin{bmatrix} \Delta \dot{\delta}_n \\ \Delta \dot{\omega}_n \end{bmatrix} = \begin{bmatrix} 0 & I \\ -M^{-1} H_n & -M^{-1} D \end{bmatrix} \begin{bmatrix} \Delta \delta_n \\ \Delta \omega_n \end{bmatrix} \tag{19}$$

In (19), $I$ is the $n \times n$ identity matrix and 0 represents a $(n \times n)$ matrix of zeros. This is a $(2n)$ set of equations representing $n$ electric angles and electric speeds of all $n$ generators.

*Supplementary Note 4: Coordinate transformation*

In power system stability analysis, one bus is appointed as the reference generator (commonly, the slack generator) and all other angles are analyzed relative to the angle of the reference generator [2], [3], [41]. Subsequently, system stability is analyzed as relative angle stability that assesses whether the angles remain relatively bounded, which does not hold true if the pace of their evolution is not homogeneous (i.e., the definition of synchronization). This is a common technique and here we borrow our analysis from [2]. From (18) it can be seen that $H$ is singular:

$$\sum_{j=1}^{n} H_{ij} = 0 \tag{20}$$

This singularity can be used for a coordinate transformation to express all electric angles as relative electric angles with respect to the reference angle. Let us suppose that the reference angle is the

electric angle of the $n$-th generator. For the coordinate transformation, one can write [2]:

$$\Delta P_i = \sum_{j=1}^{n} H_{ij}\Delta\delta_i = \sum_{j=1,j\neq n}^{n} H_{ij}\Delta\delta_i + H_{in}\Delta\delta_n \tag{21}$$

In (21), for the last term, $H_{in}\Delta\delta_n$, by leveraging the singularity property of the Laplacian it can be expressed as $H_{in}\Delta\delta_n = -\sum_{j=1,j\neq n}^{n} H_{ij}\Delta\delta_n$. Substituting it in (21) produces

$$\Delta P_i = \sum_{j=1,j\neq n}^{n} H_{ij}\ (\Delta\delta_i - \Delta\delta_n) = \sum_{j=1,j\neq n}^{n} H_{ij}\ \Delta\delta_{in} \tag{22}$$

Now, (15) can be expressed in the form of relative angles as:

$$\begin{bmatrix} \Delta P_1 \\ \Delta P_2 \\ \vdots \\ \Delta P_n \end{bmatrix} = \begin{bmatrix} H_{1,1} & H_{1,2} & \dots & H_{1,n-1} \\ H_{2,1} & H_{2,2} & \dots & H_{2,n-1} \\ \vdots & \vdots & \ddots & \vdots \\ H_{n,1} & H_{n,2} & \dots & H_{n,n-1} \end{bmatrix} \begin{bmatrix} \Delta\delta_{1,n} \\ \Delta\delta_{2,n} \\ \vdots \\ \Delta\delta_{n-1,n} \end{bmatrix} \tag{23}$$

And in compact form:

$$\Delta P_n = H_{n-1}\ \Delta\delta_{n-1} \tag{24}$$

where $H_{n-1}$ is an $n \times (n-1)$ matrix and the angles presented in $\Delta\delta_{i,n}$ are the relative angles between node $i$ and the reference angle at the reference generator $n$. Replacing (24) into (13) produces:

$$\begin{bmatrix} \Delta\dot{\delta}_{n-1} \\ \Delta\dot{\omega}_n \end{bmatrix} = \begin{bmatrix} 0 & 1_{-1} \\ -M^{-1}H_{n-1} & -M^{-1}D_n \end{bmatrix} \begin{bmatrix} \Delta\delta_{n-1} \\ \Delta\omega_n \end{bmatrix} \tag{25}$$

In (25), [0] represents a $(n \times n)$ matrix of zeros and $[1_{-1}]$ represents a matrix of identity with an extended column whose arrays are all equal to (-1). This is a $(2n-1)$ set of equations representing $(n-1)$ electric angle of all generators relative to the reference generator and $n$ actual electric speed that would include the reference generator's electric speed.

*Supplementary Note 5: Deriving the stability condition*

Here we solve the necessary condition for stable synchronization in electric power networks with heterogeneous coupling damping factors. The linear model previously developed and described in (25) can alternatively be described as:

$$\begin{bmatrix} \Delta\dot{\delta}_{i,n} \\ \Delta\dot{\omega}_i \\ \Delta\dot{\omega}_n \end{bmatrix} = \begin{bmatrix} 0 & I & -1 \\ h_i & d_i & 0 \\ h_n & 0 & d_n \end{bmatrix} \begin{bmatrix} \Delta\delta_{i,n} \\ \Delta\omega_i \\ \Delta\omega_n \end{bmatrix} \tag{26}$$

where $\Delta$ is the linear difference operator, $\delta_{i,n}$ is the vector of $(n-1)$ relative electric angles and $\omega_i$ and $\omega_n$ are absolute electric speeds expressed by a vector of $(n-1)$ and an integer value (making it a vector with a single array), respectively. [0] is a matrix of zeros, and $[I]$ is the identity matrix, each a square matrix of dimension $(n-1)$, and $[-1]$ is a matrix with dimension $1 \times (n-1)$ and all elements are (-1). $h_i = -M_i^{-1}H_{i,j}$ and $d_i = -M_i^{-1}D_i, \forall i, j = 1, 2, \cdots, (n-1)$ are diagonal matrices,

each a square matrix with dimension of $(n-1)$, with $H$, $D$, and $M$ the network interconnection Laplacian coefficients and generators damping and inertia coefficients, respectively. Finally, $h_n = -M_n^{-1} H_{i,n}, \forall i = 1, 2, \cdots, (n-1)$ is a $1 \times (n-1)$ matrix and $d_n = -M_n^{-1} D_n$ is a single array.

$$\begin{aligned}
h_i &= \begin{bmatrix} -\frac{H_{1,1}}{M_1} & -\frac{H_{1,2}}{M_1} & \dots & -\frac{H_{1,n-1}}{M_1} \\ -\frac{H_{2,1}}{M_2} & -\frac{H_{2,2}}{M_2} & \dots & -\frac{H_{2,n-1}}{M_2} \\ \vdots & \vdots & \ddots & \vdots \\ -\frac{H_{n-1,1}}{M_{n-1}} & -\frac{H_{n-1,2}}{M_{n-1}} & \dots & -\frac{H_{n-1,n-1}}{M_{n-1}} \end{bmatrix} \\
d_i &= \begin{bmatrix} -\frac{D_1}{M_1} & 0 & \dots & 0 \\ 0 & -\frac{D_2}{M_2} & \dots & 0 \\ \vdots & \vdots & \ddots & \vdots \\ 0 & 0 & \dots & -\frac{D_{n-1}}{M_{n-1}} \end{bmatrix} \\
h_n &= \begin{bmatrix} \frac{-H_{n,1}}{M_n} & \frac{-H_{n,2}}{M_n} & \dots & \frac{-H_{n,n-1}}{M_n} \end{bmatrix}
\end{aligned} \tag{27}$$

By definition of eigenvalues:

$$Jw = \lambda w \tag{28}$$

with $J$ being the system Jacobian along the solution trajectory. It can be extended as

$$\left[\begin{array}{ccc|ccc|c} 0 & \cdots & 0 & 1 & \cdots & 0 & -1 \\ \vdots & \ddots & \vdots & \vdots & \ddots & \vdots & \vdots \\ 0 & \cdots & 0 & 0 & \cdots & 1 & -1 \\ \hline -\frac{H_{1,1}}{M_1} & \cdots & -\frac{H_{1,(n-1)}}{M_1} & -\frac{D_1}{M_1} & \cdots & 0 & 0 \\ \vdots & \ddots & \vdots & \vdots & \ddots & \vdots & \vdots \\ -\frac{H_{(n-1),1}}{M_{(n-1)}} & \cdots & -\frac{H_{(n-1),(n-1)}}{M_{(n-1)}} & 0 & \cdots & -\frac{D_{(n-1)}}{M_{(n-1)}} & 0 \\ \hline -\frac{H_{n,1}}{M_n} & \cdots & -\frac{H_{n,(n-1)}}{M_n} & 0 & \cdots & 0 & -\frac{D_{(n)}}{M_{(n)}} \end{array}\right] \left[\begin{array}{c} w_1 \\ \vdots \\ w_{(n-1)} \\ \hline w_n \\ \vdots \\ w_{2(n-1)} \\ \hline w_{(2n-1)} \end{array}\right] = \lambda \left[\begin{array}{c} w_1 \\ \vdots \\ w_{(n-1)} \\ \hline w_n \\ \vdots \\ w_{2(n-1)} \\ \hline w_{(2n-1)} \end{array}\right] \tag{29}$$

This can be presented in compact form as

$$\begin{bmatrix} 0 & I & -1 \\ h_i & d_i & 0 \\ h_n & 0 & d_n \end{bmatrix} \begin{bmatrix} w' \\ w'' \\ w''' \end{bmatrix} = \lambda \begin{bmatrix} w' \\ w'' \\ w''' \end{bmatrix} \tag{30}$$

Expansion of this expression provides:

$$\begin{aligned} I \cdot w'' - \mathbf{1} w''' &= \lambda \cdot w' \\ h_i \cdot w' + d_i \cdot w'' &= \lambda \cdot w'' \\ h_n \cdot w' + d_n \cdot w''' &= \lambda \cdot w''' \end{aligned} \tag{31}$$

where $\cdot$ is the inner product operator and $\mathbf{1}$ is a matrix of all ones of $(n-1) \times 1$.

From the third equation in (31), it can be derived:

$$\begin{aligned} h_n \cdot w' + d_n \cdot w''' &= \lambda \cdot w''' \\ h_n \cdot w' &= \left(\lambda - d_n\right) \cdot w''' \\ w''' &= \left(\lambda - d_n\right)^{-1} h_n \cdot w' \end{aligned} \tag{32}$$

From the first equation in (31):

$$\begin{aligned} I \cdot w'' - w''' &= \lambda \cdot w' \\ \mathbf{1} w''' &= I \cdot w'' - \lambda \cdot w' \end{aligned} \tag{33}$$

Replacing (32) into (33) produces

$$\begin{aligned} \left(\lambda - d_n\right)^{-1} [\mathbf{1} \otimes h_n] \cdot w' &= I \cdot w'' - \lambda \cdot w' \\ \left(\lambda - d_n\right)^{-1} [\mathbf{1} \otimes h_n] \cdot w' + \lambda \cdot w' &= I \cdot w'' \\ w'' &= \left[\left(\lambda - d_n\right)^{-1} [\mathbf{1} \otimes h_n] + \lambda I\right] \cdot w' \end{aligned} \tag{34}$$

where $\otimes$ is the outer product operator.

Let us rearrange the second equation from (31):

$$\begin{aligned} h_i \cdot w' + d_i \cdot w'' &= \lambda \cdot w'' \\ h_i \cdot w' &= \left(\lambda I - d_i\right) \cdot w'' \end{aligned} \tag{35}$$

Replacing (34) in this expression produces:

$$\begin{aligned} h_i \cdot w' &= \left(\left(\lambda - d_n\right)^{-1} [\mathbf{1} \otimes h_n] + \lambda I\right) \cdot w' \\ 0 &= \left(\left(\lambda - d_n\right)^{-1} [\mathbf{1} \otimes h_n] + \lambda I\right) \cdot w' - h_i \cdot w' \\ 0 &= \left[\left(\lambda I - d_i\right) \cdot \left(\left(\lambda - d_n\right)^{-1} [\mathbf{1} \otimes h_n] + \lambda I\right) - h_i\right] \cdot w' \end{aligned} \tag{36}$$

Since $w'$ cannot be zero, then the first expression should be equal to zero. Expression (36) can be expanded to:

$$\begin{aligned} \lambda(\lambda I - d_i)(\lambda - d_n) - (\lambda - d_n) h_i + (\lambda I - d_i) \cdot [\mathbf{1} \otimes h_n] &= 0 \\ \lambda^3 I + \lambda^2(-d_i - d_n I) + \lambda(d_i d_n) - \lambda h_i + d_n h_i + \lambda[\mathbf{1} \otimes h_n] - d_i \cdot [\mathbf{1} \otimes h_n] &= 0 \\ p(\lambda) = \lambda^3 I + \lambda^2(-d_i - d_n I) + \lambda(d_i d_n - h_i + [\mathbf{1} \otimes h_n]) + (d_n h_i - d_i \cdot [\mathbf{1} \otimes h_n]) &= 0 \end{aligned} \tag{37}$$

where $\lambda$ are the eigenvalues of the system. The necessary condition for existence of eigenvalues is that this characteristic matrix is singular and thus, $det(p(\lambda)) = 0$. The existence of stable equilibria is guaranteed if the all the eigenvalues have non-positive real part. One should note that although $p(\lambda)$ is a third-order polynomial, the solution yields only a total of $(2n-1)$ eigenvalues whose eigenvectors are linearly independent for a network with $n$ generators. This is because Eq. (32) of the above procedure projects the $(2n-1)$th eigenvector into $2(n-1)$ other dimensions, creating a total of $3(n-1)$ eigenvectors. But at the end, there are only $(2n-1)$ eigenvalues whose eigenvectors are

linearly independent and the others are repetitive of the $(2n-1)$th eigenvalue and the corresponding eigenvector.

*Supplementary Note 6: Analytical derivation of stability condition for 3-generator system*

Let us consider a 3-generator system (classic 9-bus system), depicted in Supplementary Figure 10. This is a modified system to adopt the reduced-order generator models, developed based on a standardized benchmark without any change to the line parameters or network topology [3].

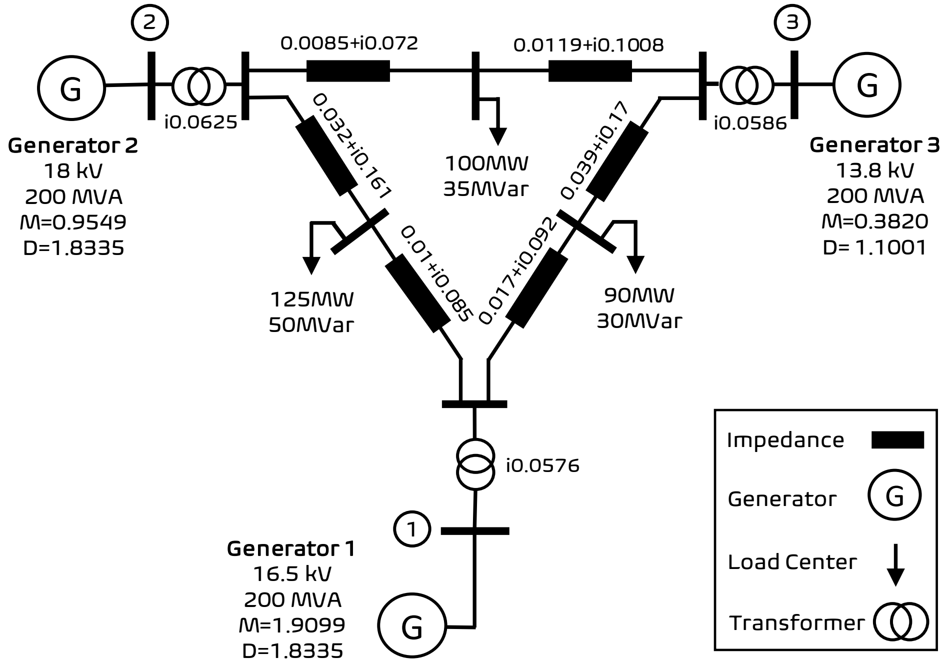


Supplementary Figure 10: One-line diagram of the 3-generator, 9-bus system, and the dynamic and network data

For this system, $n = 3$ and, let's assume gen3 as the reference generator. By definition of eigenvalues, using its Jacobian, it can be said:

$$\left[\begin{array}{cc|cc|c} 0 & 0 & 1 & 0 & -1 \\ 0 & 0 & 0 & 1 & -1 \\ \hline -\frac{H_{1,1}}{M_1} & -\frac{H_{1,2}}{M_1} & -\frac{D_1}{M_1} & 0 & 0 \\ -\frac{H_{2,1}}{M_2} & -\frac{H_{2,2}}{M_2} & 0 & -\frac{D_2}{M_2} & 0 \\ \hline -\frac{H_{3,1}}{M_3} & -\frac{H_{3,2}}{M_3} & 0 & 0 & -\frac{D_3}{M_3} \end{array}\right] \left[\begin{array}{c} a \\ b \\ \hline c \\ d \\ \hline e \end{array}\right] = \lambda \left[\begin{array}{c} a \\ b \\ \hline c \\ d \\ \hline e \end{array}\right] \tag{38}$$

with $w' = [a \quad b]^T$, $w'' = [c \quad d]^T$, and $w''' = [e]^T$, and the eigenvector $w = [w'^T \quad w''^T \quad w'''^T]^T$. These

equations can be expanded as:

$$
\begin{aligned}
c - e &= \lambda a \\
d - e &= \lambda b \\
-H_{1,1}M_1^{-1}a - H_{1,2}M_1^{-1}b - D_1M_1^{-1}c &= \lambda c \\
-H_{2,1}M_2^{-1}a - H_{2,2}M_2^{-1}b - D_2M_2^{-1}d &= \lambda d \\
-H_{3,1}M_3^{-1}a - H_{3,2}M_3^{-1}b - D_3M_3^{-1}e &= \lambda e
\end{aligned}
\tag{39}
$$

From the last equation in (39):

$$
\begin{aligned}
-H_{3,1}M_3^{-1}a - H_{3,2}M_3^{-1}b - D_3M_3^{-1}e &= \lambda e \\
-H_{3,1}M_3^{-1}a - H_{3,2}M_3^{-1}b &= (\lambda + D_3M_3^{-1})e \\
e =& (\lambda + D_3M_3^{-1})^{-1}\Big( - H_{3,1}M_3^{-1}a - H_{3,2}M_3^{-1}b\Big) \\
w''' =& \Big(\lambda - d_n\Big)^{-1} \cdot h_n \cdot w'
\end{aligned}
\tag{40}
$$

From the first two equations in (39), it can be derived:

$$
\begin{aligned}
e &= c - \lambda a \\
e &= d - \lambda b \\
\mathbf{1}w''' &= I \cdot w'' - \lambda \cdot w'
\end{aligned}
\tag{41}
$$

Replacing (40) into (41) yields:

$$
\begin{aligned}
c - \lambda a &= (\lambda + D_3M_3^{-1})^{-1}\Big( - H_{3,1}M_3^{-1}a - H_{3,2}M_3^{-1}b\Big) \\
d - \lambda b &= (\lambda + D_3M_3^{-1})^{-1}\Big( - H_{3,1}M_3^{-1}a - H_{3,2}M_3^{-1}b\Big) \\
I \cdot w'' - \lambda \cdot w' &= \Big(\lambda - d_n\Big)^{-1}[\mathbf{1} \otimes h_n] \cdot w' \\
\\
c &= (\lambda + D_3M_3^{-1})^{-1}\Big( - H_{3,1}M_3^{-1}a - H_{3,2}M_3^{-1}b\Big) + \lambda a \\
d &= (\lambda + D_3M_3^{-1})^{-1}\Big( - H_{3,1}M_3^{-1}a - H_{3,2}M_3^{-1}b\Big) + \lambda b \\
w'' &= \Big[\Big(\lambda - d_n\Big)^{-1}[\mathbf{1} \otimes h_n] + \lambda I\Big] \cdot w'
\end{aligned}
\tag{42}
$$

From the third and fourth equation in (39):

$$
\begin{aligned}
-H_{1,1}M_1^{-1}a - H_{1,2}M_1^{-1}b - D_1M_1^{-1}c &= \lambda c \\
-H_{2,1}M_2^{-1}a - H_{2,2}M_2^{-1}b - D_2M_2^{-1}d &= \lambda d \\
h_i \cdot w' + d_i \cdot w'' &= \lambda \cdot w''
\end{aligned}
\tag{43}
$$

$$
\begin{aligned}
-H_{1,1}M_1^{-1}a - H_{1,2}M_1^{-1}b &= (\lambda + D_1M_1^{-1})c \\
-H_{2,1}M_2^{-1}a - H_{2,2}M_2^{-1}b &= (\lambda + D_2M_2^{-1})d \\
h_i \cdot w' &= \Big(\lambda I - d_i\Big) \cdot w''
\end{aligned}
$$

Replacing (42) in (43) produces:

$$
\begin{aligned}
-H_{1,1}M_1^{-1}a - H_{1,2}M_1^{-1}b &= (\lambda + D_1M_1^{-1})\Big[(\lambda + D_3M_3^{-1})^{-1}\Big( - H_{3,1}M_3^{-1}a - H_{3,2}M_3^{-1}b\Big) + \lambda a\Big] \\
-H_{2,1}M_2^{-1}a - H_{2,2}M_2^{-1}b &= (\lambda + D_2M_2^{-1})\Big[(\lambda + D_3M_3^{-1})^{-1}\Big( - H_{3,1}M_3^{-1}a - H_{3,2}M_3^{-1}b\Big) + \lambda b\Big] \\
h_i \cdot w' &= \Big(\lambda I - d_i\Big) \cdot \Big[\Big(\lambda - d_n\Big)^{-1}[\mathbf{1} \otimes h_n] + \lambda I\Big] \cdot w'
\end{aligned}
\tag{44}
$$

This expression can be rearranged as:

$$
\begin{aligned}
0 &= (\lambda + D_1M_1^{-1})\Big[(\lambda + D_3M_3^{-1})^{-1}\Big( - H_{3,1}M_3^{-1}a - H_{3,2}M_3^{-1}b\Big) + \lambda a\Big] + \Big[H_{1,1}M_1^{-1}a + H_{1,2}M_1^{-1}b\Big] \\
0 &= (\lambda + D_2M_2^{-1})\Big[(\lambda + D_3M_3^{-1})^{-1}\Big( - H_{3,1}M_3^{-1}a - H_{3,2}M_3^{-1}b\Big) + \lambda b\Big] + \Big[H_{2,1}M_2^{-1}a + H_{2,2}M_2^{-1}b\Big] \\
0 &= \Big(\lambda I - d_i\Big) \cdot \Big[\Big(\lambda - d_n\Big)^{-1}[\mathbf{1} \otimes h_n] + \lambda I\Big] \cdot w' - h_i \cdot w'
\end{aligned}
\tag{45}
$$

It can be expanded as:

$$
\begin{aligned}
0 = (\lambda + D_1M_1^{-1})(\lambda + D_3M_3^{-1})^{-1}(-H_{3,1}M_3^{-1})a + (\lambda + D_1M_1^{-1})(\lambda + D_3M_3^{-1})^{-1}(-H_{3,2}M_3^{-1})b \\
\cdots + \lambda(\lambda + D_1M_1^{-1})a + \Big[H_{1,1}M_1^{-1}a + H_{1,2}M_1^{-1}b\Big] \\
0 = (\lambda + D_2M_2^{-1})(\lambda + D_3M_3^{-1})^{-1}(-H_{3,1}M_3^{-1})a + (\lambda + D_2M_2^{-1})(\lambda + D_3M_3^{-1})^{-1}(-H_{3,2}M_3^{-1})b \\
\cdots + (\lambda + D_2M_2^{-1})\lambda b + \Big[H_{2,1}M_2^{-1}a + H_{2,2}M_2^{-1}b\Big]
\end{aligned}
\tag{46}
$$

And rearranging produces:

$$
\begin{aligned}
0 =&\Big[\underbrace{(\lambda + D_1M_1^{-1})(\lambda + D_3M_3^{-1})^{-1}(-H_{3,1}M_3^{-1}) + \lambda(\lambda + D_1M_1^{-1}) + H_{1,1}M_1^{-1}}_{\psi_{11}}\Big]a \\
&\cdots + \Big[\underbrace{(\lambda + D_1M_1^{-1})(\lambda + D_3M_3^{-1})^{-1}(-H_{3,2}M_3^{-1}) + H_{1,2}M_1^{-1}}_{\psi_{12}}\Big]b \\
0 =&\Big[\underbrace{(\lambda + D_2M_2^{-1})(\lambda + D_3M_3^{-1})^{-1}(-H_{3,1}M_3^{-1}) + H_{2,1}M_2^{-1}}_{\psi_{21}}\Big]a \\
&\cdots + \Big[\underbrace{(\lambda + D_2M_2^{-1})(\lambda + D_3M_3^{-1})^{-1}(-H_{3,2}M_3^{-1}) + (\lambda + D_2M_2^{-1})\lambda + H_{2,2}M_2^{-1}}_{\psi_{22}}\Big]b \\
0 =& \Big[\underbrace{\Big(\lambda I - d_i\Big) \cdot \Big(\lambda - d_n\Big)^{-1}[\mathbf{1} \otimes h_n] + \lambda I - h_i \cdot w'}_{\psi}\Big] \cdot w'
\end{aligned}
\tag{47}
$$

In compact form, it can be written as

$$
\begin{aligned}
0 &= \psi \cdot w' \\
\psi &= \begin{bmatrix} \psi_{11} & \psi_{12} \\ \psi_{21} & \psi_{22} \end{bmatrix} \\
w' &= \begin{bmatrix} a & b \end{bmatrix}^T
\end{aligned}
\tag{48}
$$

The eigenvalues of system can be found by solving the $det(\psi) = 0$ for $\lambda$.

We have demonstrated the results for this test system using three different combinations of generation technologies and have validated them using the industry-grade full-order model, shown in Table I of the Main Manuscript. The coefficients for the reduced-order frequency response models corresponding to these case and subsequent nadir frequency and rate of change of frequency (ROCOF) are presented in Supplementary Table II, see below.

Supplementary Table II: Parameters for the cases presented in Figure 4 of the Main Manuscript

| **Case** | **Inertia Coefficient** | **Damping Coefficient** | **Nadir** | **ROCOF** |
|---|---|---|---|---|
| All SG/GFM-VSM | $(1.9099, 0.9549, 0.3820)$ | $(1.8335, 1.8335, 1.1001)$ | 59.91 | 0.276 |
| 2/3rd GFL+1/3rd SG | $(1.5915, 0.7958, 0.3183)$ | $(1.5279, 1.5279, 0.9167)$ | 59.73 | 0.376 |
| All GFM-Droop | $(0.0080, 0.0040, 0.0016)$ | $(0.3361, 0.3361, 0.2017)$ | 59.94 | 0.720 |

*Supplementary Note 7: Stability condition for uniform damping factor*

Recalling Eq. (13), let us suppose a special condition where the damping factors, $d_i = -\frac{D_i}{M_i}$, are homogeneous, constituting $d_1 = d_2 = \cdots = d_n$. The stability criteria for this special condition is developed by Motter [42] and Machowski [2]. For the sake of argument, we borrow their analysis here. The assumption of homogeneity leads to a reduced system Jacobian to $2(n-1)$ by representing electric speeds as relative electric speeds with respect to the electric speed of the reference generator. To this end, let us assume the coupling damping factor is uniform for all generators across the system

and equivalent to $(-\frac{D}{M})$. For any $i$-th generator with the $n$-th generator being the reference generator, it can be written [2]:

$$\begin{aligned}\Delta\dot{\omega}_{i,n} &= -\Big(\frac{\Delta P_i}{M_i} - \frac{\Delta P_n}{M_n}\Big) - \frac{D}{M}\Big(\Delta\omega_i - \Delta\omega_n\Big)\\ &= -\Big(\frac{\Delta P_i}{M_i} - \frac{\Delta P_n}{M_n}\Big) - \frac{D}{M}\Delta\omega_{i,n}\end{aligned} \tag{49}$$

where $\omega_{i,n} = \omega_i - \omega_n$ is the electric speed deviation from the reference machine. Substituting (49) into (13) produces

$$\begin{bmatrix}\Delta\dot{\delta}_{n-1}\\ \Delta\dot{\omega_{n-1}}\end{bmatrix} = \begin{bmatrix}0 & I\\ h & d\end{bmatrix}\begin{bmatrix}\Delta\delta_{n-1}\\ \Delta\omega_{n-1}\end{bmatrix} \tag{50}$$

where $\Delta\delta_{n-1}$ and $\Delta\omega_{n-1}$ are vectors of $(n-1)$ relative electric angle and electric speeds and $[0]$ and $[I]$ are $(n-1)\times(n-1)$ matrices of zero and identity. $d$ is a diagonal matrix of damping coefficients with all of its diagonal arrays equivalent to $(-\frac{D}{M})$ and $h$ is a $(n-1)\times(n-1)$ matrix of relative network coefficients.

To assess the stability of this system, one can calculate the eigenvalues of (50), denoted by $\lambda$ with corresponding eigenvectors of $w$. The definition of eigenvalues and eigenvectors provides [2], [42]:

$$\begin{bmatrix}0 & I\\ h & d\end{bmatrix}\begin{bmatrix}w'\\ w''\end{bmatrix} = \lambda\begin{bmatrix}w'\\ w''\end{bmatrix} \tag{51}$$

where $w = [w'^T w''^T]^T$ From (50), it can be said that $w'' = \lambda w'$ and a $h\cdot w' + dw'' = \lambda w''$ or $h\cdot w' = (\lambda - d)w''$. Replacing $w'' = \lambda w'$ into $h\cdot w' = (\lambda-d)w''$ produces $h\cdot w' = (\lambda-d)\lambda w'$ which implies $\lambda_h = \lambda^2 - d\lambda$ is the eigenvalue of the submatrix $h$. It can be rearranged as

$$\lambda^2 - d\lambda + \lambda_h = 0 \tag{52}$$

Equation (50) is equivalent to (26) under the assumption of $d_i$ homogeneity yielding a zero coupling mode $k_{d_i} = 0$ in (50). The eigenvalues of (50) can be found by solving (52) for $\lambda$ as

$$\lambda = 0.5d \pm 0.5\sqrt{d^2 + 4\lambda_h} \tag{53}$$

*Supplementary Note 8: Frequency response in power networks and hertz-sec metric*

The power network operator is concerned with continuously maintaining a stable and homogeneous system frequency across the network, 60Hz in North America and 50Hz in the rest of the world, reflecting the balance between the electric power generation and consumption. In real-world instances, this system frequency is continuously fluctuating within a small, stable equilibria as a result of slight, countless perturbations, but can be approximated as a quasi-steady state system frequency due to the small boundedness of the deviations. Any perturbation can induce transient oscillations and in a stable system these oscillations are damped out within a relatively short time following their inception. As an example, the frequency response trace from the European interconnected grid is shown in Supplementary Figure 11. The largest value of frequency excursion is called the *nadir*, which is an important value in power grid management and control because if the frequency

drops below a standard threshold – 48.5Hz in Europe [43] and 59.5Hz in North America [44] – it can trigger protective equipment that results in an interruption of power supply to some parts of the grid as a preventative measure to avoid cascading failures and blackout. Another important frequency related metric is the rate of change of frequency (ROCOF), where high values can trigger protective equipment. This metric quantifies the tangent of frequency response right after disturbance occurs and, subsequently, when the frequency starts to change. The ROCOF is measured in $\frac{Hz}{sec}$. The frequency nadir and ROCOF play a critical role in the operation of power networks and, but the individual values do not provide sufficient information about the frequency response and dynamic state of system. Therefore, to quantify and assess the frequency response, we use the hertz-sec metric [45] which is a proxy for the changes of kinetic energy induced by the disturbance. This metric integrates the absolute value of frequency deviation over its transient period. The hertz-sec, $HS$, is defined as:

$$HS = \int_{t_0}^{t_s} |f_0 - f(t)| \cdot dt \tag{54}$$

where $f_0$ and $f(t)$ are the pre-disturbance frequency (a constant) and the post-disturbance frequency (a function of time), respectively, and $t_0$ and $t_s$ are the time the frequency departs the pre-disturbance steady-state value and the time it permanently reaches the settling steady-state value, respectively.

A smaller value of this hertz-sec metric, as a proxy for reflecting smaller amount of kinetic energy, indicates a faster and more agile response, which is desired.

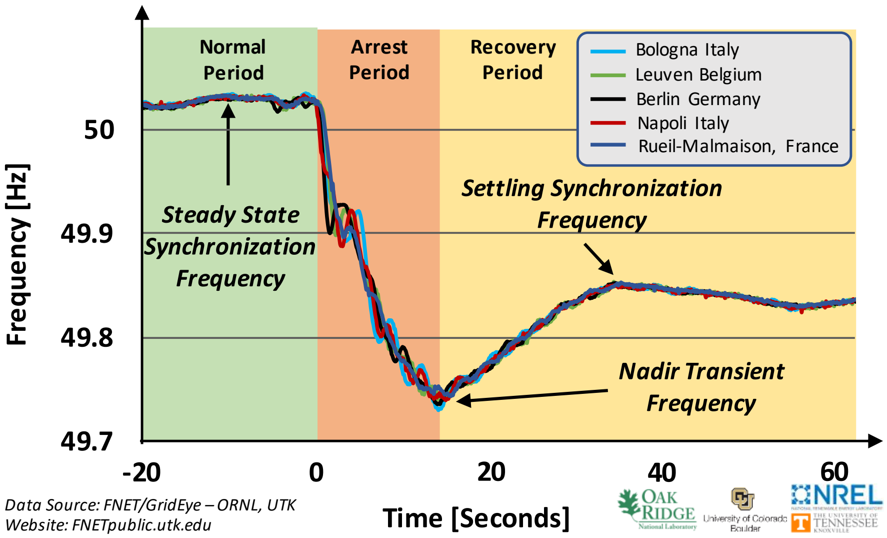


Supplementary Figure 11: Frequency response dynamic for the European synchronized continental power grid recorded by the Phasor Measurement Units (PMUs) in Italy, Belgium, Germany, and France on Jan 8, 2021: In power grid operation, the nadir frequency is the lowest point of the frequency dynamics and it is a critical value because protective equipment are set to operate if the nadir value drops below a certain threshold to avoid cascading failures and a potential major blackout. To prevent loading-shedding or disconnection of part of the grid, it is important that at dynamic state of the grid is maintained such that all times, the nadir frequency for most common small disturbances remains above this threshold level.

*Supplementary Note 9: Power network data*

We used the network data from the standard IEEE benchmarks including IEEE 9-bus [3], IEEE 30-bus [46], IEEE 39-bus [47], IEEE 57-bus [48], IEEE 118-bus [49], and IEEE 300-bus [50] systems. The steady state model for these systems is freely available as part of the MATPOWER tool [51] and a statistical description of these systems is provided in Supplementary Table III.

Supplementary Table III: Statistical description of properties of the electric power systems benchmarks considered

| **Benchmark name** | **Nodes** | **Lines** | **Generators** | **Capacity** | **Load** |
|---|---|---|---|---|---|
| IEEE 9-bus system | 9 | 9 | 3 | 0.82GW | 0.31GW |
| IEEE 30-bus system | 30 | 41 | 6 | 0.33GW | 0.19GW |
| IEEE 39-bus system | 39 | 46 | 10 | 7.36GW | 6.25GW |
| IEEE 57-bus system | 57 | 80 | 7 | 1.97GW | 1.25GW |
| IEEE 118-bus system | 118 | 186 | 54 | 9.96GW | 4.24GW |
| IEEE 300-bus system | 300 | 411 | 69 | 32.67GW | 23.52GW |

The dynamic data for some of these benchmarks does not exist. For this study, we generated synthetic dynamic data which represent real-world power system dynamics and characteristics relying on the data available from those publicly available benchmarks [3], [52]–[54]. Creation of synthetic power grids is a common practice because the real-world power system data is not always easily accessible due to legal matters, pursuant to Homeland Security Act of 2002: Critical Infrastructure Information Act [55]. Variety of dynamic power systems have been developed and documented [56]–[61], mostly extremely large systems which have application for optimization problems and power flow analysis. Our synthetic dynamic data are generated algorithmically in base cases to represent conventional power network with all generation stations equipped with synchronous generator. The range we chose is consistent with those empirically observed in the data available. The total power (MVA) capacity of generators are also randomly allocated between 100% and 250% of their total power dispatch determined by the power-flow equations to account for reserve margin. Then we validated by perturbing all generators, one generator at a time, and monitored the dynamic response of all generators that are interconnected to the network to ensure they are stable and their characteristics and response match what is commonly observed in power systems.

To perturb the system and emulate the load switching, we subjected the electric angle of generators, $\delta$ to a step change that replicates a change of the loading condition and subsequently, we monitored the electric speed, $\omega$. Because we applied Kron reduction which is a common technique in power network synchronization studies, the load nodes are eliminated and there is no other mean to directly switch loads, other than switching the $\delta$ of generators in a sustained fashion. System frequency can be measured as the electric speed of generators, $\Delta\omega = \Delta\dot{\delta}$, as $\Delta f = \Delta\omega(2\pi)^{-1} + f_{ss}$ with $f_{ss} = 60Hz$ (in the United States, 50 Hz in Europe), and $(\Delta\delta_0, \Delta\omega_0) = (0, 0)$, assuming the frequency departs from an equilibrium when disturbed.

## Supplementary References

[1] H.-D. Chiang, *Direct methods for stability analysis of electric power systems: theoretical foundation, BCU methodologies, and applications.* John Wiley & Sons, 2011.

[2] J. Machowski, J. W. Bialek, and J. R. Bumby, *Power system dynamics: stability and control.* John Wiley & Sons, 2008.

[3] P. W. Sauer and M. A. Pai, *Power system dynamics and stability*, vol. 101. Prentice hall Upper Saddle River, NJ, 1998.

[4] R. W. Kenyon, "PyPSCAD." original-date: 2020-11-09T20:50:43Z.

[5] R. W. Kenyon, A. Sajadi, A. Hoke, and B.-M. Hodge, "Open-source pscad grid-following and grid-forming inverters and a benchmark for zero-inertia power system simulations," in *2021 IEEE Kansas Power and Energy Conference (KPEC)*, pp. 1–6, IEEE, 2021.

[6] S. D'Arco, J. A. Suul, and O. B. Fosso, "A," *Electric Power Systems Research*, vol. 122, pp. 180–197, 2015.

[7] U. Markovic, J. Vorwerk, P. Aristidou, and G. Hug, "Stability analysis of converter control modes in low-inertia power systems," in *2018 IEEE PES Innovative Smart Grid Technologies Conference Europe (ISGT-Europe)*, pp. 1–6, IEEE, 2018.

[8] M. Rasheduzzaman, J. A. Mueller, and J. W. Kimball, "An accurate small-signal model of inverter-dominated islanded microgrids using $dq$ reference frame," *IEEE Journal of Emerging and Selected Topics in Power Electronics*, vol. 2, no. 4, pp. 1070–1080, 2014.

[9] M. Rasheduzzaman, J. A. Mueller, and J. W. Kimball, "Reduced-order small-signal model of microgrid systems," *IEEE Transactions on Sustainable Energy*, vol. 6, no. 4, pp. 1292–1305, 2015.

[10] H.-P. Beck and R. Hesse, "Virtual synchronous machine," in *2007 9th International Conference on Electrical Power Quality and Utilisation*, pp. 1–6, IEEE, 2007.

[11] Y. Chen, R. Hesse, D. Turschner, and H.-P. Beck, "Dynamic properties of the virtual synchronous machine (visma)," *Proc. Icrepq*, vol. 11, pp. 755–759, 2011.

[12] T. Kerdphol, M. Watanabe, Y. Mitani, D. Turschner, and H.-P. Beck, "Stability assessment of multiple virtual synchronous machines for microgrid frequency stabilization," in *2020 IEEE Power & Energy Society General Meeting (PESGM)*, pp. 1–5, IEEE, 2020.

[13] Y. Chen, R. Hesse, D. Turschner, and H.-P. Beck, "Investigation of the virtual synchronous machine in the island mode," in *2012 3rd IEEE PES Innovative Smart Grid Technologies Europe (ISGT Europe)*, pp. 1–6, IEEE, 2012.

[14] Y. Chen, R. Hesse, D. Turschner, and H.-P. Beck, "Improving the grid power quality using virtual synchronous machines," in *2011 international conference on power engineering, energy and electrical drives*, pp. 1–6, IEEE, 2011.

[15] T. Kerdphol, F. S. Rahman, M. Watanabe, Y. Mitani, D. Turschner, and H.-P. Beck, "Enhanced virtual inertia control based on derivative technique to emulate simultaneous inertia and damping properties for microgrid frequency regulation," *IEEE Access*, vol. 7, pp. 14422–14433, 2019.

[16] R. Ofir, U. Markovic, P. Aristidou, and G. Hug, "Droop vs. virtual inertia: Comparison from the perspective of converter operation mode," in *2018 IEEE International Energy Conference (ENERGYCON)*, pp. 1–6, IEEE, 2018.

[17] U. Markovic, N. Früh, P. Aristidou, and G. Hug, "Interval-based adaptive inertia and damping control of a virtual synchronous machine," in *2019 IEEE Milan PowerTech*, pp. 1–6, IEEE, 2019.

[18] U. Markovic, Z. Chu, P. Aristidou, and G. Hug, "Lqr-based adaptive virtual synchronous machine for power systems with high inverter penetration," *IEEE Transactions on Sustainable Energy*, vol. 10, no. 3, pp. 1501–1512, 2018.

[19] R. Hesse, D. Turschner, and H.-P. Beck, "Micro grid stabilization using the virtual synchronous machine (visma)," in *Proceedings of the International Conference on Renewable Energies and Power Quality (ICREPQ'09), Valencia, Spain*, pp. 15–17, 2009.

[20] Y. Chen, R. Hesse, D. Turschner, and H.-P. Beck, "Comparison of methods for implementing virtual synchronous machine on inverters," in *International conference on renewable energies and power quality*, vol. 1, pp. 414–424, 2012.

[21] Q.-C. Zhong and G. Weiss, "Static synchronous generators for distributed generation and renewable energy," in *2009 IEEE/PES Power Systems Conference and Exposition*, pp. 1–6, IEEE, 2009.

[22] Q.-C. Zhong, P.-L. Nguyen, Z. Ma, and W. Sheng, "Self-synchronized synchronverters: Inverters without a dedicated synchronization unit," *IEEE Transactions on power electronics*, vol. 29, no. 2, pp. 617–630, 2013.

[23] S. Rubino, A. Mazza, G. Chicco, and M. Pastorelli, "Advanced control of inverter-interfaced generation behaving as a virtual synchronous generator," in *2015 IEEE Eindhoven PowerTech*, pp. 1–6, IEEE, 2015.

[24] M. Blau and G. Weiss, "Synchronverters used for damping inter-area oscillations in two-area power systems," in *Int. Conf. on Renew. Energies and Power Quality (ICREPQ)*, 2018.

[25] K. Sakimoto, Y. Miura, and T. Ise, "Stabilization of a power system with a distributed generator by a virtual synchronous generator function," in *8th International Conference on Power Electronics-ECCE Asia*, pp. 1498–1505, IEEE, 2011.

[26] J. Liu, Y. Miura, and T. Ise, "Dynamic characteristics and stability comparisons between virtual synchronous generator and droop control in inverter-based distributed generators," in *2014 International Power Electronics Conference (IPEC-Hiroshima 2014-ECCE ASIA)*, pp. 1536–1543, IEEE, 2014.

[27] P. Rodriguez, I. Candela, and A. Luna, “Control of pv generation systems using the synchronous power controller,” in *2013 IEEE Energy Conversion Congress and Exposition*, pp. 993–998, IEEE, 2013.

[28] W. Zhang, A. Luna, I. Candela, J. Rocabert, and P. Rodriguez, “An active power synchronizing controller for grid-connected power converters with configurable natural droop characteristics,” in *2015 IEEE 6th International Symposium on Power Electronics for Distributed Generation Systems (PEDG)*, pp. 1–7, IEEE, 2015.

[29] P. R. CORTÉS, J. I. C. García, J. R. Delgado, and R. Teodorescu, “Virtual controller of electromechanical characteristics for static power converters,” Mar. 6 2014. US Patent App. 14/001,853.

[30] P. R. Cortés, J. I. C. Garcia, J. R. Delgado, and R. Teodorescu, “Synchronous power controller for a generating system based on static power converters,” Oct. 20 2015. US Patent 9,166,508.

[31] L. Shi and M. Crow, “Power and energy society general meeting-conversion and delivery of electrical energy in the 21st century,” *IEEE, Pittsburgh*, 2008.

[32] M. Van Wesenbeeck, S. De Haan, P. Varela, and K. Visscher, “Grid tied converter with virtual kinetic storage,” in *2009 IEEE Bucharest PowerTech*, pp. 1–7, IEEE, 2009.

[33] Y. Hirase, K. Abe, K. Sugimoto, and Y. Shindo, “A grid-connected inverter with virtual synchronous generator model of algebraic type,” *Electrical Engineering in Japan*, vol. 184, no. 4, pp. 10–21, 2013.

[34] Y. Hirase, K. Sugimoto, K. Sakimoto, and T. Ise, “Analysis of resonance in microgrids and effects of system frequency stabilization using a virtual synchronous generator,” *IEEE Journal of Emerging and Selected Topics in Power Electronics*, vol. 4, no. 4, pp. 1287–1298, 2016.

[35] S. D’Arco, J. A. Suul, and O. B. Fosso, “Control system tuning and stability analysis of virtual synchronous machines,” in *2013 IEEE Energy Conversion Congress and Exposition*, pp. 2664–2671, IEEE, 2013.

[36] F. Mandrile, E. Carpaneto, and R. Bojoi, “Grid-tied inverter with simplified virtual synchronous compensator for grid services and grid support,” in *2019 IEEE Energy Conversion Congress and Exposition (ECCE)*, pp. 4317–4323, IEEE, 2019.

[37] F. Mandrile, E. Carpaneto, and R. Bojoi, “Grid-feeding inverter with simplified virtual synchronous compensator providing grid services and grid support,” *IEEE Transactions on Industry Applications*, vol. 57, no. 1, pp. 559–569, 2020.

[38] M. Awal and I. Husain, “Unified virtual oscillator control for grid-forming and grid-following converters,” *IEEE Journal of Emerging and Selected Topics in Power Electronics*, 2020.

[39] V. Mallemaci, F. Mandrile, S. Rubino, A. Mazza, E. Carpaneto, and R. Bojoi, “A comprehensive comparison of virtual synchronous generators with focus on virtual inertia and frequency regulation,” *Electric Power Systems Research*, vol. 201, p. 107516, 2021.

[40] T. Jouini, U. Markovic, and D. Groß, “Control and operation of a grid with 100% converter-based devices-new options for existing system services and needs for new system services,” *Deliverable (D3. 3) Report from EU-funded MIGRATE project*, 2018.

[41] P. Kundur, N. J. Balu, and M. G. Lauby, *Power system stability and control*, vol. 7. McGraw-hill New York, 1994.

[42] A. E. Motter, S. A. Myers, M. Anghel, and T. Nishikawa, “Spontaneous synchrony in power-grid networks,” *Nature Physics*, vol. 9, no. 3, pp. 191–197, 2013.

[43] ENTSO-E, “Technical background and recommendations for defence plans in the continental europe synchronous area,” 2010.

[44] NERC, “Standard bal-003-1 — frequency response and frequency bias setting,” 2013.

[45] R. W. Kenyon and B. Mather, “Quantifying transmission fault voltage influence and its potential impact on distributed energy resources,” in *2018 IEEE Electronic Power Grid (eGrid)*, pp. 1–6, IEEE, 2018.

[46] R. Christie, “Ieee 30-bus system,” 1993.

[47] T. Athay, “Ieee 39-bus system,” 1979.

[48] R. Christie, “Ieee 57-bus system,” 1993.

[49] R. Christie, “Ieee 118-bus system,” 1962.

[50] M. Adibi, “Ieee 300-bus system,” 1993.

[51] R. Zimmerman, C. Murillo-Sanchez, and D. Gan, “Matpower: A matlab power system simulation package 2006,” *See http://pserc. cornell. edu/matpower*, 2009.

[52] P. C. Krause, O. Wasynczuk, S. D. Sudhoff, and S. Pekarek, *Analysis of electric machinery and drive systems*, vol. 2. Wiley Online Library, 2002.

[53] V. Vittal and R. Treinen, “Ieee 145-bus system,” 1987.

[54] T. Xu, A. B. Birchfield, K. S. Shetye, and T. J. Overbye, “Creation of synthetic electric grid models for transient stability studies,” in *The 10th Bulk Power Systems Dynamics and Control Symposium (IREP 2017)*, pp. 1–6, 2017.

[55] G. M. Stevens, "Homeland security act of 2002: Critical infrastructure information act," LIBRARY OF CONGRESS WASHINGTON DC CONGRESSIONAL RESEARCH SERVICE, 2003.

[56] A. B. Birchfield, T. Xu, and T. J. Overbye, "Power flow convergence and reactive power planning in the creation of large synthetic grids," *IEEE Transactions on Power Systems*, vol. 33, no. 6, pp. 6667–6674, 2018.

[57] H. Li, J. H. Yeo, A. Bornsheuer, and T. J. Overbye, "The creation and validation of load time series for synthetic electric power systems," *IEEE Transactions on Power Systems*, 2020.

[58] E. Schweitzer, T. Xu, A. B. Birchfield, A. Scaglione, T. J. Overbye, R. J. Thomas, and Z. Wang, "Towards operational validation: Mapping power system inputs to operating conditions," in *2018 Power Systems Computation Conference (PSCC)*, pp. 1–7, IEEE, 2018.

[59] A. B. Birchfield, T. Xu, K. M. Gegner, K. S. Shetye, and T. J. Overbye, "Grid structural characteristics as validation criteria for synthetic networks," *IEEE Transactions on power systems*, vol. 32, no. 4, pp. 3258–3265, 2016.

[60] T. Xu, A. B. Birchfield, and T. J. Overbye, "Modeling, tuning, and validating system dynamics in synthetic electric grids," *IEEE Transactions on Power Systems*, vol. 33, no. 6, pp. 6501–6509, 2018.

[61] S. Babaeinejadsarookolaee, A. Birchfield, R. D. Christie, C. Coffrin, C. DeMarco, R. Diao, M. Ferris, S. Fliscounakis, S. Greene, R. Huang, *et al.*, "The power grid library for benchmarking ac optimal power flow algorithms," *arXiv preprint arXiv:1908.02788*, 2019.
